# A Cross-Sectional Study of Factors Predicting the Duration of the Efficacy of Viscosupplementation in Knee Osteoarthritis

**DOI:** 10.3390/jcm13071949

**Published:** 2024-03-27

**Authors:** Charles Rapp, Feriel Boudif, Charlotte Bourgoin, Anne Lohse, Thierry Conrozier

**Affiliations:** 1 Department of Rheumatology, Hôpital Nord Franche-Comté, 90400 Belfort, France; cj-rapp@outlook.fr (C.R.); feriel.boudif@hnfc.fr (F.B.); anne.lohse@hnfc.fr (A.L.); 2 Clinical Research Unit, Hôpital Nord Franche-Comté, 90400 Belfort, France; charlotte.bourgoin@hnfc.fr

**Keywords:** hyaluronic acid, viscosupplementation, knee, osteoarthritis, effectiveness, predictors

## Abstract

**Background:** An advanced radiological stage and obesity are predictive of poorer and shorter responses to viscosupplementation in patients with knee osteoarthritis (OA). Very little is known regarding the impact of other factors such as sport practice, comorbidities, or anatomical features of OA. **Methods:** This study aimed to investigate patients’ and OA characteristics associated with the duration of the effectiveness (DE) of viscosupplementation in patients with knee OA. It was a cross-sectional, single-centre clinical trial in patients with knee OA treated with intra-articular (IA) hyaluronic acid (HA) injection(s) within the previous 3 years. The investigators collected data regarding demographic and radiographic features (Kellgren–Lawrence grade and involved knee compartments), dosing regimen (single or repeat injections), the presence and volume of joint effusion, previous or concomitant IA corticosteroid injection, the number of previous viscosupplementations, and comorbidities. Patients completed a questionnaire including the self-assessment of DE (the number of weeks during which viscosupplementation was effective on symptoms), the activity level (sedentary, active, or athletic), and the level of sport activity (light, moderate, or intensive). Predictors of the DE were studied in bivariate and multivariate analyses. **Results:** In total, 105 patients (149 knees) were analysed (62% women, mean age 66.1 ± 13.2 years, mean BMI 27.5 ± 7.5 kg/m^2^). The mean DE was 48.2 ± 24.8 weeks. In bivariate analysis, the predictors of a shorter DE were BMI > 27.5 kg/m^2^, more than three previous viscosupplementations, Kellgren–Lawrence grade 4, sedentary patients, and multicompartmental involvement. In the multivariate analysis, four independent factors remained associated with a shorter DE: BMI > 27.5 kg/m^2^, multicompartmental knee involvement, number of viscosupplementations >3, and sedentary lifestyle. A statistically significant association between a longer DE and arterial hypertension was found, suggesting a beneficial effect of certain antihypertensive medications. **Conclusions:** This study confirms that being overweight significantly reduces the duration of the effectiveness of viscosupplementation. It also shows that viscosupplementation is more lastingly effective in unicompartmental OA and among active or athletic patients. The duration of effectiveness decreases when the treatment is repeated more than three times.

## 1. Introduction

Knee osteoarthritis (OA) is a common condition, with an estimated prevalence of 7.6% among the general population, i.e., around 600 million people worldwide [1]. Although not directly life-threatening, knee OA can have a major impact on a patient’s quality of life and indirectly affects mortality through disability, obesity, and cardiovascular problems caused by a lack of physical activity and the use of non-steroidal anti-inflammatory drugs (NSAIDs) [2].

OA is characterised by the degradation of hyaline articular cartilage, followed by damage to all joint tissues (subchondral bone, synovium, and capsule), leading to pain and functional impairment that can even lead to severe disability. To date, there is no treatment that can restore the cartilage or even halt its deterioration. The only definitive treatment is knee replacement, with problems inherent in surgery, its cost, anaesthesia, and the limited lifespan of the prosthesis, which often requires a repeat operation one or two decades later. Conservative treatments for knee OA include a combination of pharmacological and non-pharmacological modalities [3,4,5], none of which are considered to be highly effective. Among the pharmacological methods, viscosupplementation through the intra-articular (IA) injection(s) of hyaluronic acid (HA) has the highest effect size (0.63–95%, CrI: 0.39 to 0.88) [6]. The concept of viscosupplementation was introduced in the 1990s by EA. Balazs [7] hypothesised that injecting high-molecular-weight HA intra-articularly would improve joint function by restoring the viscoelastic properties of the synovial fluid (SF). It has since been demonstrated that HA has not only a lubricating and shock-absorbing effect but also other anti-inflammatory, analgesic, antiapoptotic, and antidegenerative properties [8]. Viscosupplementation is a symptomatic treatment for pain in knee OA, recommended by several learned societies [3,4,5] when first-line treatments (analgesics and NSAIDs) are not sufficiently effective. The safety of viscosupplementation is excellent; the relative risk of an adverse reaction versus saline is 1.01 (95%CI 0.96–1.07 *p* = 0.6) [9], making it the treatment of choice for a population that is often elderly and compromised by pre-existing conditions. However, although it is widely used worldwide and has produced good results in clinical practice [10], its actual level of efficacy remains controversial. This is why the Osteoarthritis Research Society International (OARSI) [11] and the American College of Rheumatology [12] do not recommend viscosupplementation in all situations and prefer, as first-line treatments, NSAIDs (topical and oral), corticosteroid IA injections, and non-pharmacological methods (weight loss, physical exercise, balance, and proprioception training). HA injections are only recommended conditionally if first-line treatments fail or are unsuitable. 

All guidelines agree that the treatment of knee osteoarthritis should be personalised and tailored to each patient’s individual needs and profile in order to achieve the most effective outcome possible [3,4,5,11,12]. We have previously shown that obesity and the radiographic severity of OA were independent factors for a poorer response to viscosupplementation [13,14]. The aim of this new study was to identify the predictive factors of the duration of viscosupplementation efficacy under “real-life” conditions, by not only studying the role of radiological and demographic characteristics but also including many other factors in the analysis, such as lifestyle habits, pre-existing conditions, and treatments of comorbidities, as well as current and previous treatments for OA. We chose to describe the duration of response to treatment based on patients’ clinical experience to reflect current daily clinical practice as closely as possible.

## 2. Patients and Methods

PRESAGE (ClinicalTrials.gov Identifier: NCT04988698) was a single-centre, cross-sectional study, conducted in 2022 and 2023 at the Hôpital Nord Franche-Comté (HNFC, Belfort, France), aimed at studying the factors predicting the duration of the effectiveness (DE) of viscosupplementation in patients suffering from knee OA. 

The study received approval from the Comité de Protection des Personnes Sud EST III (ID-CRB No. 2021-A00773-38). The trial was conducted in accordance with good clinical practice and the Declaration of Helsinki.

### 2.1. Patient Inclusion/Exclusion Criteria

All adult outpatients who were referred to the rheumatology department of the Nord Franche-Comté Hospital for more than 2 months (minimum time to feel the effectiveness of viscosupplementation) and less than 3 years (the time beyond which the reliability of the answer is questionable) after having been treated with viscosupplementation for symptomatic OA of the knee and who agreed to participate were included in the study. Patients who were unable to complete the questionnaire due to cognitive problems or language barriers were excluded from the study, as were patients who had not given their consent and those treated with viscosupplementation for a reason unrelated to knee OA.

### 2.2. Study Progression

During a routine consultation, investigators gave patients an information document in order to obtain informed consent. The investigators collected demographic data (age, sex, weight, height, and body mass index (BMI)) and radiographic data on the knees, including the Kellgren–Lawrence grades modified by Felson [15] and the compartments affected by OA (i.e., patellofemoral [PF], medial tibiofemoral [MTF], and lateral tibiofemoral [LTF]). The radiographs were centrally read by the same experienced investigator (T.C.). 

The investigators also gathered data on the treatment regimen performed, namely single or repeated injections: single-injection procedures were performed exclusively with cross-linked HA (HappyCross^®^, Synvisc-One^®^, Durolane^®^, and Hymovis^®^). Patients who had been injected with linear HA (Happyvisc^®^, Arthrum^®^, and Synolis-VA^®^) systematically underwent a procedure consisting of 3 injections separated by 7 days.

The presence and volume of any joint effusion (the amount of SF removed prior to injection) on the day HA was administered was recorded, as was the number of previous viscosupplementations, previous IA corticosteroid injections, or IA steroid injections concomitant with viscosupplementation.

Finally, patients completed a questionnaire including the following information:

- Self-assessment of the duration of treatment efficacy (DE = number of weeks during which viscosupplementation was effective on symptoms);
- The degree of satisfaction with the treatment on a numerical scale from 0 to 10;
- Activity level: sedentary, active, or athletic;
- Physical activity practised and intensity: light, moderate, or intense.

For the statistical analysis, the physical activity practised was divided into two categories: “low impact” on the knee joint and “high impact” comprising sports with a moderate and/or high impact on the knee according to Buckwalter and Jane’s classification [16]. The low-impact group includes walking, hiking, Pilates, swimming, downhill skiing, and occasional cycling. The high-impact group includes moderate-to-intense running, trail running, skating, cross-country skiing, tennis and football, and intense cycling. Based on these considerations, the strain on the knees was classified as low, light, normal, or heavy.

### 2.3. Statistical Analysis

The data were analysed using the R++ software (“R++, l’essentiel” for Windows, Version 1.6.15, Toulouse, France). A descriptive statistical analysis was carried out on the population, expressed as headcount, mean, and standard deviation for quantitative variables, and percentage and confidence interval for qualitative variables.

For the bivariate analysis, we used Welch’s *t*-test, Student’s *t*-test, Mann–Whitney U test, or one-way ANOVA, as appropriate. The primary endpoint was the DE self-reported by the patient. The significance level was set at 5%. A multivariate analysis was then performed using linear regression according to factors with a *p*-value < 0.1 found in the bivariate analysis.

## 3. Results

**Figure 1:**
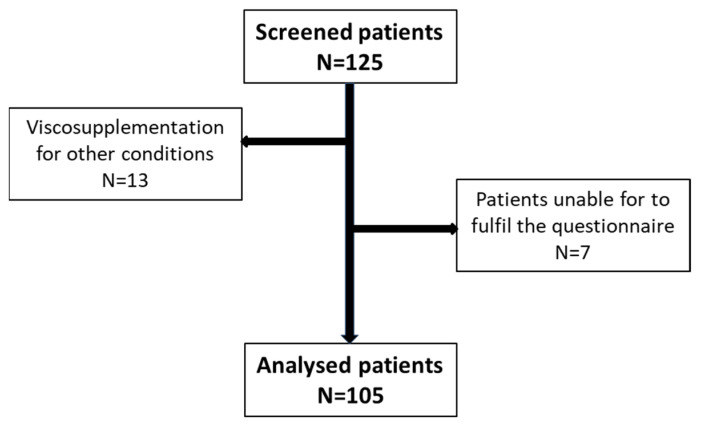
Flowchart.

After inclusion, 105 patients completed the questionnaire. The flowchart is presented in Figure 1. The general characteristics of patients are shown in Table 1. The mean age was 66.1 ± 13.2 years, and the mean BMI was 27.5 ± 7.5 kg/m^2^. Our population included a larger proportion of women (62%) and retired people (57%).

For the statistical analysis of the primary endpoint, we studied this population by knee treated, with a total of 149 knees studied, the details of which are provided in Table 2. The male/female distribution remained similar, as did the proportion of athletes. 

The early radiological grades, 1–2, accounted for 43% of the knees, compared with 57% for grades 3 and 4. The mean and median radiological grade was 3 (i.e., 41 knees). Osteoarthritis affected only one compartment (TF or PF) in 60% of cases (TF or PF), while in the other 40%, OA affected two or three compartments. At the time of injection, 59 patients were taking painkillers and/or NSAIDs. All except 1 subject reduced their analgesic consumption, and at the time of consultation, only 22 subjects were still taking analgesics (*n* = 18) or NSAIDs (*n* = 4). Twenty-six patients were taking symptomatic slow-acting drugs for OA (SYSADOA). Out of the 149 injections, 133 were performed with cross-linked HA using a single-injection procedure. Only 16 injections were performed with linear hyaluronic acid over 3 injections. In our rheumatology department, we are used to only using cross-linked HA for single-injection protocols and linear HA for repeated injections, regardless of the OA anatomical severity. On the day of viscosupplementation, most knees (68%) presented an effusion, mostly of a small volume (mean 1.98 mL ± 4.47 mL (range 0.1–50)), requiring concomitant IA long-lasting corticosteroid injection in only 10 cases.

The mean DE of viscosupplementation in the whole population was 48.2 + 24.8 weeks (median 48 weeks). The detailed characteristics of the population are shown in Table 2.

**Figure 2:**
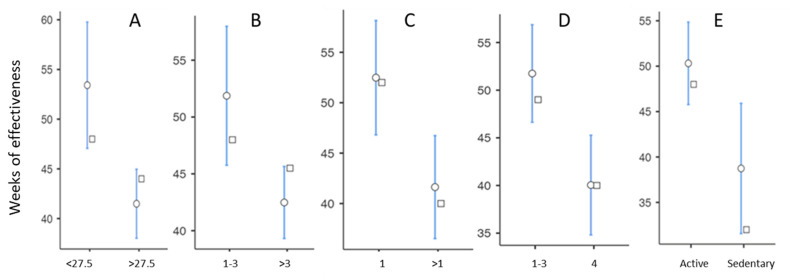
Duration of the viscosupplementation effectiveness according to (**A**) body mass index (kg/m^2^); (**B**) number of previous viscosupplementations; (**C**) number of involved knee compartments; (**D**) Kellgren–Lawrence grade; (**E**) lifestyle (◦ = mean; □ = median).

In a bivariate analysis of the factors associated with DE, we found a strong influence of BMI, with a mean DE of 53.4 ± 29.7 weeks in patients with a BMI < 27.5 kg/m^2^ compared with 41.16 ± 14.36 weeks in patients with a BMI > 27.5 kg/m^2^ (*p* = 0.002) (Figure 2A). Regarding the number of viscosupplementations performed, we found a significant reduction in the DE from the fourth cycle of injections onwards (Figure 2B). 

The location of OA also had a statistically significant impact. OA with the best DE was the isolated MTF involvement, which had an average DE of 57.3 ± 31.8 weeks. More generally, unicompartmental forms had a DE that was 11 weeks longer on average than multicompartmental forms (*p* = 0.01) (Figure 2C). In the unicompartmental forms, subjects with MTF OA had a longer DE than those with PF and LTF OA, close to statistical significance (*p* = 0.10).

Radiographic grade 4 was associated with a decrease in the DE of around 12 weeks, with a mean DE of 40.0 ± 17.9 weeks, compared with 51.8 ± 26.6 for grades < 4 (Figure 2D). There was no statistically significant difference in the DE between grades 1, 2, and 3. Active patients had a mean DE of 50.3 ± 25.5 weeks, 12 weeks longer than sedentary patients, whose mean DE was 38.7 ± 19.0 weeks (Figure 2E). It should be noted that there was no statistical difference in the DE between patients practising sports and active patients not practising sports or between patients practising high-impact and low-impact sports. 

There was no statistically significant difference in the DE according to the duration of symptoms, the use of SYSADOAs or NSAIDs, the presence and volume of effusion, dose regimen, or the concomitant injection of corticosteroids. It should be noted that the 10 corticosteroid injections were carried out in patients treated with a single injection and that the DE was not statistically different between subjects receiving IA CS and those who did not (44.2 ± 42.4 versus 49.4 ± 24.2 weeks; *p* = 0.54). Women had a moderately shorter DE, but this was not statistically significant (*p* = 0.138). The presence of one or more pre-existing conditions did not affect the DE. Surprisingly, however, we found a borderline statistically significant association between treated arterial hypertension and the DE, with a longer DE in hypertensive patients compared with non-hypertensive patients (53.1 ± 31.3 versus 45.4 ± 19.8 weeks; *p* = 0.068). Details of these data are summarised in Table 3. 

In the multivariate analysis, we identified four independent factors associated with a shorter DE: BMI > 27.5 kg/m^2^, knee multicompartment damage, the number of viscosupplementations >3, and a sedentary lifestyle. We found a statistically significant more prolonged DE in patients treated for high blood pressure (*p* < 0.001). It is important to emphasise that, in the multivariate analysis, the radiological grade was no longer associated with the DE (*p* = 0.22) (Table 4).

## 4. Discussion

In this cross-sectional study, several independent factors were shown to influence the DE of viscosupplementation in patients with knee OA: being overweight, the number of involved knee compartments, the number of previous viscosupplementations, lifestyle, the treatment for arterial hypertension, and to a lesser extent the radiological grade. This study confirms once again that being overweight is associated with a shorter duration of the efficacy of viscosupplementation, as it has already been described in obesity [13,14]. In our study, we found that the DE decreased even in cases where the patient was moderately overweight, from a BMI of 27.5 kg/m^2^. It is well demonstrated that an increase in body fat is associated with higher levels of proinflammatory cytokines, increased production of adipokines with deleterious effects on articular cartilage, the upregulation of proteolytic enzymes such as matrix metalloproteinases and agrecanases, and increased production of reactive oxygen species, all of which are involved in the pathophysiology of OA [16]. This reinforces the importance of weight loss in patients suffering from OA and could be one explanation for the potentially reduced efficacy of this treatment in North America, where the prevalence of obesity is around 32% [17], compared with 17% in France [18]. Wang et al. reported poorer efficacy in patients aged over 65 years [19], which we did not find in terms of the duration of efficacy. However, although 41% of our patient population was under 65 years of age, there were very few patients under 50 (8%). A case–control study could reveal a hidden difference in our study. Another explanation could be that older patients are more satisfied with a more modest effect, as has already been pointed out [20].

Although we found that advanced radiological grade was a factor in a reduced response to viscosupplementation in the bivariate analysis, we did not find any statistical difference in the multivariate analysis between the DE and radiological grade 4, unlike Eymard et al. [13], Altman et al. [21] and Perruchet et al. [22]. This could be explained by the fact that our results were obtained under conditions of daily clinical practice, without any exclusion criteria (no upper limit for BMI and radiological grade), unlike the studies of Eymard et al. [13] and Altman et al. [21], which were prospective randomised controlled trials with strict inclusion/exclusion criteria. Radiological grade 4 patients included in our analysis were likely those with mild-to-moderate symptoms, while subjects with more severe symptoms were referred to a surgeon for joint replacement. As a result, we were unable to evaluate the DE of viscosupplementation in patients lost to follow-up because they were referred to a surgeon for total knee replacement. Although we found a longer DE for viscosupplementation in unicompartmental OA, particularly MTF OA, the DE in multicompartmental damage remained satisfactory (average 41.6 weeks).

We found no difference between a triple-injection dosing regimen with linear HA and a single-injection approach with cross-linked HA, demonstrating that cross-linking is a valid method that allows for a single-injection dosing regimen [23,24,25], which is beneficial for the patient and the doctor’s schedule and reduces the indirect costs [26] and carbon footprint.

It is interesting to note that we observed a constant DE over time until the fourth treatment cycle, beyond which the DE decreased slightly but still remained satisfactory. The presence of an effusion at the time of viscosupplementation did not affect the DE. However, only three knees from our population had an effusion of more than 10 mL. It is interesting to underline that the only patient with a large effusion of 50 mL reported an efficacy of only 34 weeks, despite being active and having a BMI < 27.5, two factors contributing to a long DE. We did not observe that an injection of corticosteroids concomitant with the injection of HA significantly increased the DE of the latter. However, the limited number of patients who received a corticosteroid injection was too small to draw definitive conclusions. In terms of physical activity, we found no reduction in the DE in athletic patients, including those who performed activities that put a lot of strain on the knees. However, these patients probably require more demanding treatment in terms of joint function, with a potentially lower DE than non-athletic patients. Although the DE was not significantly different in athletic and active patients, we found a significantly shorter DE in sedentary patients than in active or athletic patients, including in the multivariate analysis. This justifies the value of physical activity in the management of knee OA, as already established [27,28], and not restricting the exercise of patients suffering from OA. Concerning the practice of “extreme” sports such as very long-distance running (only one patient practised endurance trail running in our population), which is very popular at the moment and is likely to be common practice among patients with OA in a few years’ time, it is advisable to be cautious, and a specific case–control study could be useful given the limited number of people with OA currently practising these sports. However, clinical trials aimed at assessing the benefits of high-intensity training (HIT) have shown that HIT improves not only knee OA symptoms and physical functioning but also aerobic capacity, muscle strength, and quality of life with minimal or no adverse events [29].

Surprisingly, we also found a longer DE in patients treated for arterial hypertension. As it is unlikely that hypertension has a positive effect on OA symptoms, the most logical hypothesis is that certain antihypertensive medications could have a beneficial effect. We did not record the medication taken by the patients, but in terms of frequency, the most frequently prescribed medications in France are angiotensin II receptor blockers and diuretics followed by beta-blockers, calcium channel blockers, and angiotensin-converting enzyme (ACE) inhibitors [30]. Several experimental studies [31,32,33,34,35,36,37,38,39,40] have shown that certain antihypertensives have potentially beneficial effects on OA, whether through anti-inflammatory, antioxidant, analgesic, or even antidegenerative effects. Our work is therefore in line with the literature on the potential chondroprotective effect of certain antihypertensive medications, but as we did not record the medications, we cannot draw any conclusions in this respect. Other studies specifically designed for this purpose need to be carried out to confirm or refute the beneficial role of antihypertensive treatment on symptoms of knee OA.

Our study has obviously several strengths and limitations. The main strength is that it evaluates the DE of viscosupplementation in real-life conditions, without selecting age, BMI, or radiographic grade. In addition, all the patients evaluated were treated according to the recommended procedures, i.e., a single injection of cross-linked product or a triple injection of linear product with a 7-day interval between each injection, in a centre specialising in the treatment of OA patients and injected by highly experienced senior rheumatologists, with a centralised reading of the radiographs. Finally, to our knowledge, this is the first study to look at such a large number of parameters, in particular patients’ lifestyle habits. The decision to choose “patient self-assessment of DE” as the primary endpoint was a pragmatic one. Although the notion of effectiveness is subjective and varies from subject to subject, depending on their expectations, it corresponds to clinical practice where composite scores (e.g., Western Ontario and McMaster Universities Arthritis score (WOMAC) and Knee Injury and Osteoarthritis Outcome (KOOS)) [41] are rarely used in routine consultations. However, efficacy is well correlated with patient satisfaction and a reduction in the patient’s global assessment and WOMAC score [42].

One of the weaknesses of our study is that it is monocentric. The results therefore reflect the habits of a single centre. However, as pointed out above, the investigators had considerable experience in viscosupplementation. In addition, the cross-sectional nature of the study means that we performed no quantified assessment of changes in disability and pain over time. However, as mentioned above, the patient’s overall assessment of his or her condition has been shown to be well correlated with these values [42]. We also had certain information biases. Having been unable to obtain a reliable assessment of analgesic consumption, it was not possible for us to measure the impact of the latter on the DE of viscosupplementation. The data on physical activity and intensity were reported by the patient, and we had no objective data to corroborate them. We also had a patient recruitment bias, with some patients being systematically called back at 1 year, resulting in the DE being capped at 52 weeks, whereas some patients had opted not to return until the pain had returned. Finally, we were unable to assess the number of patients who met the inclusion criteria but were not seen again, either because of treatment failure or because the efficacy was still in progress and did not justify a return visit.

In conclusion, despite its limitations, our work provides new useful information concerning the predictors of the success of viscosupplementation, as one of the doctor’s duties is to accurately inform patients about the treatment offered to them. While it confirms the harmful influence of an increased BMI, the novelty of this study lies in the fact that the negative influence of BMI appears as early as 27.5 kg/m^2^, which corresponds to moderately overweight patients. It also shows that viscosupplementation is more effective in unicompartmental OA, while radiological grade 4 appears to have a lesser influence than previously published. Other points worth highlighting are the reduction in the duration of efficacy with repeated cycles of injections and the absence of any difference between patients practising sports and those who regularly use their lower limbs in their everyday activities. Once again, this confirms the value of physical activity in the overall management of OA of the knee. Finally, contrary to what we might have thought, our results do not show a shorter DE in patients practising high-impact sports than in athletic people engaged in activities that place less strain on the knees. Prospective, longitudinal large-scale trials are necessary to confirm these data.

## Figures and Tables

**Table 1:** Patient characteristics at the time of injection (*n* = 105).

**Age ± SD (range) (years)**	66.1 ± 13.2 (22–90)
% <65 years	41%
% <50 years	8%
% <40 years	<2%
**Sex**	
Female	66 (62%)
Male	39 (38%)
**Body mass index (kg/m^2^)**	
Median	26.8
Mean ± SD (range)	27.5 ± 5.55 (18.3–43.7)
**Professional status**	
Working	35 (33%)
Unemployed	10 (10%)
Retired	60 (57%)
**Sport practice**	
Yes *n* (%)	46 (43.8%)
No *n* (%)	59 (56.2%)
**Patients taking drugs for knee OA (%)**	68%
Analgesics	45%
Non-steroidal anti-inflammatory drugs	13%
Glucosamine	17%
Chondroitin	9%

**Table 2:** Characteristics of the knees treated with viscosupplementation (VS) at time of injection (*n* = 149).

**Duration of VS effectiveness (weeks)**	
Mean ± SD	48.2 ± 24.8
Median (range)	48 (0–156)
**Disease duration *n* (%)**	
>5 years	85 (57%)
>1 and ≤5 years	46 (31%)
≤1 year and >6 months	10 (6.7%)
≤6 months	7 (4.7%)
Missing data	1
**Kellgren–Lawrence grade**	
Median (range)	3 (1–4)
Mean ± SD	2.8 ± 1.1
Grade 1–2 *n* (%)	63 (43%)
Grade 3 *n* (%)	41 (27%)
Grade 4 *n* (%)	45 (30%)
**Involved compartments**	
Patellofemoral	83 (57%)
Isolated patellofemoral	29 (20%)
Tibiofemoral	63 (43%)
Medial tibiofemoral	47 (32%)
Unicompartmental	89 (61%)
Multicompartmental	57 (39%)
Missing data	3 (2%)
**Dosing regimen**	
Cross-linked HA single injection	133 (89%)
Linear HA repeated injections	16 (11%)
**Time since VS (weeks)**	
Mean ± SD	54.4 ± 24.4
Median (range)	52 (25–160)
**Number of VS**	
Mean ± SD	3.54 ± 2.44
Median (range)	3 (1–14)
**Synovial effusion at injection—*n* (%)**	101 (67.8%)
**Volume of effusion (mL)**	
Mean ± SD	1.98 ± 4.47
Median (range)	1 (0–50)
**Corticosteroid injection—*n* (%)**	10 (6.7%)

**Table 3:** Predictors of the duration of effectiveness of viscosupplementation: bivariate analysis.

	DE (Weeks) (Mean ± SD) (Range)	95%CI
BMI		
<27.5 (*n* = 84)	53.4 ± 29.7 (4–156)	47.0–59.9
>27.5 (*n* = 65)	41.16 ± 14.36 (0–65)	38.0–45.0
Welch’s *t*-test: 3.23 *p*: 0.002		
Sex		
Female (*n* = 92)	45.5 ± 18.6 (16–124)	41.7–49.4
Male (*n* = 57)	52.5 ± 32.2 (0–156)	44.0–61.1
Welch’s *t*-test: −1.50 *p*: 0.138		
Physical activity		
Active (*n* = 122)	50.3 ± 25.5 (0–156)	45.7–54.9
Sedentary(*n* = 27)	38.7 ± 19.0 (16–80)	31.2–46.3
Mann–Whitney U test: 1083 *p*: 0.005		
Kellgren–Lawrence grade		
Grade < 4 (*n* = 104)	51.8 ±26.6 (0–156)	46.6–56.9
Grade 4 (*n* = 45)	40.0 ± 17.9 (4–100)	34.7–45.4
Student’s *t*-test: 2.70 *p*: 0.008		
Number of viscosupplementations		
1–3 (*n* = 91)	51.9 ± 29.7 (0–156)	45.7–58.1
>3 (*n* = 58)	42.5 ± 12.3 (16–73)	39.2–45.7
Welch’s *t*-test: 2.67 *p*: 0.008		
Treatment for arterial hypertension		
No	45.4 ± 19.8 (0–124)	41.3–49.4
Yes	53.1 ± 31.3 (16–156)	44.6–61.5
Student’s *t*-test: −1.84 *p*: 0.068		
Analgesic treatment		
No (*n* = 82)	50.8 ± 27.7 (4–156)	44.7–56.9
Yes (*n* = 67)	45.0 ± 20.6 (0–108)	40.0–50.1
Student’s *t*-test: 1.41 *p*: 0.160		
Number of involved compartments		
1 (*n* = 89)	52.5 ± 27.3 (0–156)	46.7–58.2
2 or 3 (*n* = 57)	41.6 ± 19.7 (4–109)	36.4–46.8
Student’s *t*-test: 2.60 *p*: 0.010		
Involved compartments		
PF (*n* = 29)	48.3 ± 22.7 (16–124)	39.6–56.9
LTF (*n* = 13)	44.4 ± 13.6 (20–65)	36.2–52.6
MTF (*n* = 47)	57.3± 31.8 (0–156)	48.0–66.6
MTF + PF(*n* = 36)	43.4 ± 18.8 (18–109)	37.0–49.7
LTF + PF (*n* = 10)	32.9 ± 12.8 (16–52)	23.7–42.1
MTF + LTF (*n* = 3)	48.7 ± 7.0 (42–56)	31.2–66.1
LTF + LTF + PF (*n* = 8)	42.1 ± 30.9 (4–108)	16.3–67.9
ANOVA: 2.59 *p* = 0.046		
Treatment with SYSADOA		
No (*n* = 111)	48.4 ± 28.1 (0–156)	43.1–53.7
Yes (*n* = 38)	47.6 ± 10.6 (26–73)	44.1–51.1
Welch’s *t*-test: 0.257 *p*: 0.797		
Treatment with NSAIDs		
No (*n* = 130)	48.7 ± 25.9 (0–156)	44.2–53.2
Yes (*n* = 19)	44.9 ± 15.3 (26–80)	37.5–52.3
Mann–Whitney U test: 1103 *p*: 0.453		
Comorbidities		
Non (*n* = 46)	44.6 ± 16.9 (4–124)	
Yes (*n* = 103)	49.8 ± 27.6 (0–156)	
Student’s *t*-test: −1.18 *p*: 0.240		
SF effusion		
Yes (*n* = 101)	46.3 ± 22.5 (0–155)	41.8–50.7
No (*n* =48)	52.3± 28.9 (4–156)	43.9–60.7
Student’s *t*-test: −1.39 *p*: 0.168		
Dosing regimen		
Single injection (*n* = 133)	48.9 ± 25.8 (0–156)	44.5–53.3
3 injections (*n* = 16)	42.3 ± 14.4 (15–65)	34.6–50
Student’s *t*-test: 1.01 *p*: 0.316		
Corticosteroid injection		
No (*n* = 138)	48.6 ± 23.4 (4–156)	44.6–52.5
Yes (*n* = 10)	44.2 ± 42.4 (0–155)	13.9–74.5
Student’s *t*-test: 503 *p*: 0.153		
Sport practice		
Yes (*n* = 66)	47.4 ± 23.8 (0–156)	41.6–53.3
No (*n* = 83)	48.8 ± 25.8 (16–155)	43.2–54.5
Student’s *t*-test: −0.339 *p*: 0.735		
No sport (*n* = 83)	49.4 ± 26.1 (16–53.5)	43.2–54.2
Sports with low impact (*n* = 51)	46.3 ± 20.9 (4–124)	40.4–52.2
Sports with high impact (*n* = 15)	51.4 ± 32.3 (0–156)	33.5–68.3
ANOVA: 0.347 *p*: 0.706		
Sportive versus active patients		
Athletic (*n* = 66)	47.4 ± 23.8 (0–156)	41.6–53.3
Active but not athletic (*n* = 56)	53.7 ± 27.3 (18–155)	46.4–61.0
Student’s *t*-test: −1.35 *p*: 0.179		
Knee stress		
High (*n* = 24)	48 ± 25.98 (0–156)	37.2–59.1
Normal/moderate (*n* = 103)	50.31 ± 25.14 (4–155)	45.4–55.2
Low (*n* = 22)	38.5 ± 20.425 (16–80)	29.4–47.6
Kruskal–Wallis test: 7.95 *p*: 0.019		

DE: duration of effectiveness; BMI: body mass index; SYSADOA: symptomatic slow-acting drugs for OA; NSAIDs: non-steroidal anti-inflammatory drugs; SF: synovial fluid.

**Table 4:** Predictors of DE of viscosupplementation: multivariate analysis.

Predictors	Estimation	Standard Error	t	*p*
Intercept	59.75	3.38	17.68	<0.001
BMI:				
>27.5 versus <27.5	−14.92	4.15	−3.60	<0.001
Number of involved compartments	−9.13	3.96	−2.30	0.023
≥2 versus 1				
K-L grade:				
Grade 4 versus grade < 4	−5.49	4.46	−1.23	0.220
Number of VS:				
>3 versus 1 to 3	−9.35	3.94	−2.37	0.019
Arterial hypertension:				
Yes versus No	16.39	4.23	3.88	<0.001
Physical activity:				
Sedentary versus Active	−11.75	5.25	−2.24	0.027

DE: duration of effectiveness; BMI: body mass index; K-L: Kellgren–Lawrence; VS: viscosupplementation.

## Data Availability

Data are available at the Clinical Research Unit of Hôpital Nord Franche-Comté (URC HNFC), Trevenans, France.
